# Outcomes of Upper Gastrointestinal Bleeding at United States Teaching and Non-teaching Hospitals: A National Inpatient Sample Analysis

**DOI:** 10.7759/cureus.61793

**Published:** 2024-06-06

**Authors:** Mohamad Sharbatji, Prabhu Anand Sachin, Ravinuthala Abhishek, Saeed Ali, Asad Ur Rahman

**Affiliations:** 1 Internal Medicine, AdventHealth Orlando, Orlando, USA; 2 Internal Medicine, University of Illinois Chicago, Chicago, USA; 3 Gastroenterology, Cleveland Clinic Florida, Weston, USA

**Keywords:** upper gastrointestinal bleed, major outcomes, gastric variceal bleeding, national inpatient sample database, hospital teaching status

## Abstract

Background/aims: Studies have varied results regarding the impact of the teaching and non-teaching status of hospitals on the outcomes for hospitalized patients with upper gastrointestinal bleeding (UGIB). To evaluate these outcomes, we conducted a retrospective cohort study using the 2014 National Inpatient Sample (NIS) database.

Methods: We included all adult patients who were admitted with the principal diagnosis of UGIB. Patients admitted to rural and urban non-teaching hospitals were classified as non-teaching, whereas those admitted to urban teaching hospitals were classified as teaching. The main outcomes of interest were in-hospital mortality, percentage of patients requiring inpatient endoscopy, and endoscopic therapy, packed red blood cell (PRBC) transfusion, length of stay (LOS), and total hospitalization charges.

Results: The study included 132,085 (97%) with nonvariceal UGIB (NVUGIB) and 4,200 (3%) with variceal UGIB (VUGIB). Of them, 62% were managed at teaching hospitals. Compared with admitted patients at non-teaching hospitals, patients with nonvariceal UGIB admitted at teaching hospitals had similar adjusted in-hospital mortality rates (adjusted odds ratio (OR): 0.97, 95% confidence interval (CI): 0.79-1.19), inpatient endoscopy rates (OR: 0.98, 95% CI: 0.91-1.1), and early endoscopy rates (within 24 hours) (OR: 0.98, 95% CI: 0.91-1.1) and lower PRBC transfusion rates (OR: 0.87, 95% CI: 0.79-0.97) but higher endoscopic therapy rates (OR: 1.3, 95% CI: 1.2-1.4), length of stay (mean increase of 0.43 days) (P<0.01), and total hospital charges (mean increase of $4,369) (P<0.01). Patients with variceal UGIB had similar adjusted in-hospital mortality rates (OR: 1.2, 95% CI: 0.61-2.3), inpatient endoscopy rates (OR: 0.97, 95% CI: 0.67-1.4), early endoscopy rates (within 24 hours) (OR: 0.97, 95% CI: 0.67-1.4), endoscopic therapy rates (OR: 2.5, 95% CI: 0.54- 11.2), and total hospital charges (P=0.45), and lower PRBC transfusion rates (OR: 0.63, 95% CI: 0.45-0.88) but higher length of stay (mean increase of 0.69 days) (P=0.02).

Conclusions: Patients with nonvariceal UGIB treated at US teaching hospitals and non-teaching hospitals have similar mortality, rates of in-hospital endoscopy, and early endoscopy, but teaching hospitals have higher rates of in-hospital therapeutic endoscopy, length of stay, and total hospital charges. There was no difference in any of the outcomes for variceal gastrointestinal (GI) bleeding treated at teaching hospitals compared with those treated at non-teaching hospitals, except for length of stay, which was higher among patients admitted to teaching hospitals compared to those admitted to non-teaching hospitals.

## Introduction

Upper gastrointestinal bleeding (UGIB) is a medical emergency that requires immediate medical attention and in most cases admission to the hospital for further workup such as esophagogastroduodenoscopy (EGD). In the United States, hospitals can be classified based on academic status and affiliation with medical schools into two large categories: teaching hospitals and non-teaching hospitals. Teaching hospitals are places where medical students, resident physicians, subspecialties fellows, and healthcare trainees receive their education under close supervision from an experienced academic physician. Teaching hospitals are usually large establishments and in general regarded as the best hospitals [1].

Hospital teaching status has been shown to have a significant impact on clinical outcomes in both medical and surgical care [1,2]. Recent evidence suggests that hospital teaching status is associated with lower mortality in patients with high admission risk [3,4]. One of the most common and life-threatening conditions observed in an inpatient setting is upper gastrointestinal bleeding (UGIB). A variety of factors have been shown to affect outcomes. For example, "the weekend effect" has notably been used to describe the phenomenon of increased adverse outcomes in UGIBs admitted over the weekend, likely because endoscopy suites are functioning with limited staff during weekends, leading to delays in endoscopic management [5].

Some studies have found worse outcomes, such as mortality and risk of mechanical ventilation/shock, in cases with nonvariceal gastrointestinal bleeding (NVUGIB) treated at a teaching hospital compared to a non-teaching hospital [6]. Similar findings have been demonstrated in cases with variceal bleeds where mortality rate, length of stay (LOS), and cost were higher at teaching hospitals [7]. A criticism of these findings, however, is that some teaching hospitals are associated with universities, which often see a population with higher acuity. In many cases, such university medical systems are publicly funded and, therefore, represent the safety net for the local area's most underserved and highest-risk population. Findings on the association between hospital teaching status and clinical outcomes in UGIB patients appear inconclusive. As a result, it is important to further characterize and quantify the role of teaching status in clinical management and overall outcomes. The goal of this study is to determine how various clinical outcome markers are affected by hospital teaching status, specifically in the setting of both NVUGIB and variceal upper gastrointestinal bleeding (VUGIB).

## Materials and methods

Study design and database description

This is a retrospective analysis of a large national database. We used the National Inpatient Sample (NIS) database, which is one of the largest publicly maintained databases by the Agency for Healthcare Research and Quality as a part of the Healthcare Cost and Utilization Project and is derived from billing data submitted by hospitals across the United States to statewide data organizations. It covers 97% of the US population and approximates a 20% stratified sample of discharges from US community hospitals. The dataset is weighted to obtain national estimates. In 2014, the NIS was coded using the International Classification of Disease, Ninth Revision, Clinical Modification/Procedure Coding System (ICD-9-CM/PCS). The database can be accessed at https://www.hcup-us.ahrq.gov [8].

Study population

This study included all adults hospitalized with UGIB in the United States in 2014. The ICD-9-CM system does not have a unified code for UGIB. Therefore, ICD-9 codes of previously published studies with similar patient populations were used [9]. Only patients with a principal ICD-9-CM diagnosis for UGIB were included in the study. Patients with a discharge code unique for hemorrhage of the gastrointestinal (GI) tract without further indications of the bleeding site such as unspecified hemorrhage of the GI tract (578.9), acute posthemorrhagic anemia (285.1), or blood in stool/melena (578.1) must be accompanied by another ICD-9-CM code suggestive of a possible source of blood loss from the upper GI tract before inclusion. Study cohorts were classified into variceal and nonvariceal UGIB based on ICD-9-CM codes [10,11].

Statistical analysis

Statistical analysis was conducted using STATA/BE 17.0 (StataCorp, College Station, TX) to account for weights in the stratified survey design of the NIS database. The weights were considered in the statistical estimating process by incorporating the variable for strata, clusters, and weight to discharge in the NIS database. Descriptive statistics were provided, which included the mean (standard error) for continuous variables and count (percentage) for categorical variables. The Rao-Scott design-adjusted chi-square test, which takes the stratified survey design into account, examined the association between demographic variables, individual comorbidities profile, and hospital characteristics and main outcomes that included the following: in-hospital mortality, inpatient endoscopy defined as endoscopy that occurred during the index admission, and endoscopic therapy rate, packed red blood cell (PRBC) transfusion rate, length of stay, and total hospitalization charges [12]. The difference in the median age, median length of stay (LOS), and median total hospital charges were tested by contrast using F-statistics from a weight regression model. Multivariable logistics regression was utilized to calculate the odds for the other outcomes. The adjusted odds ratio (OR) with a 95% confidence interval (CI) was reported. All analytical results were significant when P values were ≤0.05.

## Results

Patient characteristics

There were 35 million discharges included in the NIS 2014, of which 136,285 met the study inclusion criteria: 132,085 (97%) with nonvariceal UGIB and 4,200 (3%) with variceal UGIB. Of the study population, 84,539 (62%) were admitted to a teaching hospital. Compared with patients admitted to a non-teaching hospital, patients admitted at a teaching hospital were less likely to be female, White, or insured by Medicare. They also had lower median annual income (below $63,000) and Charlson Comorbidity Index scores (0, 1, and 2) as shown in Table 1 and Table 2. Although the differences are statistically different, the absolute differences were small. Hospital characteristics were also statistically different; however, the absolute difference was not shown in Table 1 and Table 2.

**Table 1:** Characteristics of patients with upper gastrointestinal bleeding admitted in the United States in 2014 No.: Numbers, %: percentage, y: year, $: United States dollar

Characteristics	Teaching hospital admission	Non-teaching hospital admission	P value
Patients, no. (%)	84,539 (62)	51,746 (38)	
Female, no. (%)	37,000 (44)	24,000 (46)	<0.01
Race/ethnicity, no. (%)			
White	54,950.3 (65)	38,809.5 (75)	<0.01
African-American	15,217 (18)	5,174.6 (10)	<0.01
Hispanic	9,299.2 (11)	4,915.87 (9.5)	<0.01
Asian or Pacific Islander	1,690.7 (2)	931.4 (1.8)	<0.01
Native American	760.8 (0.9)	569.2 (1.1)	<0.01
Other	2,113.4 (2.5)	1,034.9 (2)	<0.01
Median age, y (25th-75th percentile)	63.7	65.6	<0.01
Charlson Comorbidity Index score, no. (%)			
0 (no comorbidity)	17,753.2 (21)	11,901.6 (23)	<0.01
1	16,062.4 (19)	11,384.1 (22)	<0.01
2	11,835.5 (14)	8,279.4 (16)	<0.01
3 or more ( multiple comorbidities)	38,042.6 (45)	20,180.9 (39)	<0.01
Median annual income in patient's zip code, $, no. (%)			
$1-$38,999	26,207.1 (31)	16,041.2 (31)	<0.01
$39,000-$47,999	21,134.8 (25)	16,041.2 (31)	<0.01
$48,000-$62,900	19,444 (23)	11,901.5 (23)	<0.01
$63,000 or more	16,907.8 (20)	8,279.4 (16)	<0.01
Insurance type, no. (%)			
Medicare	50,468 (59)	33,117.4 (64)	<0.01
Medicaid	13,686.2 (16)	7,244.4 (14)	<0.01
Private	16,252.4 (19)	8,796.8 (17)	<0.01
Uninsured	5,132.3 (6)	2,846 (5)	<0.01
Endoscopy within 24 hours after admission, no. (%)	46,618.7 (54.5)	28,977.8 (56)	0.0789

**Table 2:** Hospital characteristics for upper gastrointestinal bleed admissions in the United States in 2014 No.: number, %: percentage

Hospital characteristics	Teaching hospital	Non-teaching hospital	P value
Hospital region, no. (%)			
Northeast	18,598.6 (22)	6,727 (13)	<0.01
Midwest	19,444 (23)	10,866.6 (21)	<0.01
South	31,279.4 (37)	21,215.9 (41)	<0.01
West	15,217 (18)	12,936.5 (25)	<0.01
Hospital bed size, no. (%)			
Small	17,753.19 (21)	6,727 (13)	<0.01
Medium	26,207 (31)	15,523.8 (30)	<0.01
Large	40,578.7 (48)	29,495.2 (57)	<0.01
Admissions in July	6,763.1 (8)	4,139.68 (8)	0.6858

In-hospital mortality based on admission to teaching hospitals

The in-hospital mortality rate for patients with total UGIB was 1.9%. Hospital teaching status was not an independent predictor of overall in-hospital mortality compared with hospitals with non-teaching status on both univariable and multivariable analysis (adjusted OR: 0.96, 95% confidence interval (CI): 0.79-1.16). When adjusting only for the factors associated with correlation with mortality defined as P<0.2 on univariate analysis (older age, higher Charlson Comorbidity Index score, admissions occurred other than the month of July, non-White racial groups, and insurance type (uninsured and Medicaid)), a similar result was retrieved (adjusted OR: 0.95, 95% CI: 0.79-1.15).

In-hospital mortality rates for nonvariceal and variceal UGIB were 1.8% and 6.2%, respectively (Table 3). Admission at a teaching hospital was not an independent predictor of mortality for patients with nonvariceal UGIB (adjusted OR: 0.97, 95% CI: 0.79-1.19) or with variceal UGIB (adjusted OR: 1.2, 95% CI: 0.61-2.3).

**Table 3:** Overall in-hospital mortality for admitted patients with UGIB to teaching and non-teaching hospitals in 2014 UGIB: upper gastrointestinal bleeding, NVUGIB: nonvariceal upper gastrointestinal bleeding, VUGIB: variceal upper gastrointestinal bleeding, No.: numbers, %: percentage

	Total death	Teaching hospital admission	Non-teaching hospital admission
All UGIB, no. (%)	2,589/136,285 (1.9)	1,630/84,539 (1.9)	970/51,746 (1.9)
NVUGIB, no. (%)	2,339/132,085 (1.8)	1,455/81,893 (1.8)	884/50,192 (1.7)
VUGIB, no. (%)	260/4,200 (6.2)	175/2,646 (6.6)	84/1,554 (5.4)

In-hospital packed red blood cell (PRBC) transfusion rates based on admission to teaching hospitals

For patients with total UGIB, the in-hospital PRBC transfusion rate of at least one unit of PRBC was 44% of admissions. Patients admitted to teaching hospitals were less likely to receive blood transfusions. Admission to teaching hospitals was a predictor of a lower chance of receiving blood transfusion after adjusting for both patient- and hospital-level confounders (adjusted OR: 0.86, 95% CI: 0.78-0.96). Among patients with nonvariceal UGIB, the in-hospital PRBC transfusion rate of at least one unit of PRBC was 43% of admissions. Patients admitted to teaching hospitals were less likely to receive blood transfusions. Admission to teaching hospitals was a predictor of a lower chance of receiving blood transfusion after adjusting for both patient- and hospital-level confounders (adjusted OR: 0.87, 95% CI: 0.78-0.97). For patients with variceal UGIB, the in-hospital PRBC transfusion rate of at least one unit of PRBC was 52% of admissions. Patients admitted to teaching hospitals were less likely to receive blood transfusions. Admission to teaching hospitals was a predictor of a lower chance of receiving blood transfusion after adjusting for both patient- and hospital-level confounders (adjusted OR: 0.63, 95% CI: 0.45-0.88).

Inpatient endoscopy rates and time to endoscopy based on admission to teaching hospitals

Inpatient endoscopy was performed in 78% of admissions with any UGIB, and endoscopic therapy was done in 15% of admissions. Teaching hospital admissions were less likely to undergo an inpatient endoscopy; however, after adjusting for both patient- and hospital-level confounders, the difference disappears (adjusted OR: 0.98, 95% CI: 0.91-1.1). Teaching hospital admission was a predictor of endoscopy intervention (adjusted OR: 1.3, 95% CI: 1.2-1.4) (Table 3). In nonvariceal UGIB, endoscopy was performed in 79% of admissions, and endoscopy therapy was done in 16% of admissions. Teaching hospital admission had a similar chance of undergoing inpatient endoscopy, even after adjusting for both patient- and hospital-level confounders (adjusted OR: 0.98, 95% CI: 0.91-1.1). Teaching hospital admission was a predictor of endoscopy intervention (adjusted OR: 1.3, 95% CI: 1.2-1.4) (Table 4). The median time from admission to endoscopy was 1.4 days for nonvariceal UGIB. Time to endoscopy was similar in teaching hospitals compared to non-teaching hospitals (difference in timing to EGD: 0.02 days) (95% CI: -0.1-0.1). Teaching hospital admission was not an independent predictor of early endoscopy for patients with nonvariceal UGIB on multivariable analysis (adjusted OR: 0.98, 95% CI: 0.91-1.1)

**Table 4:** Odds ratio of inpatient EGD or therapeutic EGD for all UGIB, NVUGIB, and VUGIB EGD: esophagogastroduodenoscopy, UGIB: upper gastrointestinal bleeding, NVUGIB: nonvariceal upper gastrointestinal bleeding, VUGIB: variceal upper gastrointestinal bleeding, OR: odds ratio, CI: confidence interval

	All UGIB		NVUGIB		VUGIB	
	Crude OR (95% CI)	Adjusted OR (95% CI)	Crude OR (95% CI)	Adjusted OR (95% CI)	Crude OR (95% CI)	Adjusted OR (95% CI)
Upper endoscopy	0.93 (0.87-99)	0.98 (0.91-1.1)	0.93 (0.87-1)	0.98 (0.91-1.1)	0.81 (0.60-1.1)	0.90 (0.65-1.2)
Endoscopic therapy	1.2 (1.1-1.3)	1.3 (1.2-1.4)	1.23 (1.13-1.3)	1.3 (1.2-1.4)	1.91 (0.61-6)	2.5 (0.54-11.2)

The rate of inpatient endoscopy was 36% for variceal UGIB. The median time from admission to endoscopy was 0.8 days. Teaching hospital admission had a similar time to endoscopy compared to non-teaching hospital admission (difference in timing from admission to EGD: 0.06 days) (95% CI: -0.1-0.2). Admission at teaching hospitals was not an independent predictor of early endoscopy for patients with variceal UGIB (adjusted OR: 0.97, 95% CI: 0.67-1.4). Admission at teaching hospitals was not an independent predictor of either in-hospital endoscopy performance (adjusted OR: 0.90, 95% CI: 0.65-1.2) or endoscopic therapy (adjusted OR: 2.5, 95% CI: 0.54-11.2) on multivariable analysis.

Hospital length of stay based on admission to teaching hospitals

Hospital length of stay based on teaching hospital status for patients with total UGIB, nonvariceal UGIB, and variceal UGIB are shown in Table 4. Nonvariceal UGIB admissions at teaching hospitals had slightly longer hospital stays by 0.43 days (P<0.01) compared with admissions at non-teaching hospitals. Nonvariceal UGIB admissions that underwent early endoscopy (within 24 hours from admission) have a significantly shorter adjusted length of stay (median: 3.69 days) compared with those that did not (median: 4.78 days), regardless of hospital teaching status (P<0.01) (Table 5). Length of stay was shorter for patients who underwent an early endoscopy compared with those who did not, with an adjusted decrease in length of stay by 0.97 days (P<0.01). Variceal UGIB admissions at teaching hospitals had slightly longer hospital stays by 0.69 days (P=0.01) compared with admissions at non-teaching hospitals. Length of stay was similar for patients who underwent an early endoscopy compared with those who did not after adjusting for patient and hospital confounder (P<0.2) (Table 5).

**Table 5:** Unadjusted median length of stay for all UGIB, NVUGIB, and VUGIB UGIB: upper gastrointestinal bleeding, NVUGIB: nonvariceal upper gastrointestinal bleeding, VUGIB: variceal upper gastrointestinal bleeding

	Teaching hospital admission	Non-teaching hospital admission
All UGIB, days (25th-75th percentile)	4.39 (4.31-4.47)	3.89 (3.81-3.96)
NVUGIB, days (25th-75th percentile)	4.37 (4.29-4.46)	3.88 (3.8-3.95)
VUGIB, days (25th-75th percentile)	4.89 (4.52-5.25)	4.18 (3.79- 4.58)

Total hospital charges based on admission to teaching hospitals

The mean total hospitalization charges for each admission were $38,381 for all UGIB. After adjusting for patient- and hospital-level confounders, patients admitted to teaching hospitals had significantly higher total charges (additional $4,276 per admission) (P<0.01) compared with those admitted to non-teaching hospitals. Patients who underwent early endoscopy (done within 24 hours from admission) had significantly lower total charges (by $3,238) (P<0.01) compared to those who did not, regardless of teaching hospital status. For nonvariceal UGIB, the mean total hospitalization charges for each admission were $37,989. After adjusting for patient- and hospital-level confounders, patients admitted to teaching hospitals had significantly higher total charges (additional $4,369 per admission) (P<0.01) compared with those admitted to non-teaching hospitals. Patients who underwent early endoscopy (done within 24 hours from admission) had significantly lower total charges (by $3,404) (P<0.01) compared to those who did not, regardless of teaching hospital status. For variceal UGIB, the mean total hospitalization charges for each admission were $50,809. After adjusting for patient- and hospital-level confounders, patients admitted to teaching hospitals had similar total charges (P=0.45) compared with those admitted to non-teaching hospitals. Patients who underwent early endoscopy also had similar total charges (P=0.3) compared to those who did not, regardless of teaching hospital status (Table 6).

**Table 6:** Median length of stay (days) based on timing of endoscopy UGIB: upper gastrointestinal bleeding, NVUGIB: nonvariceal upper gastrointestinal bleeding, VUGIB: variceal upper gastrointestinal bleeding

	Endoscopy within 24 hours of admission	Endoscopy after 24 hours of admission	P value	Difference in length of stay (days)
All UGIB	3.72 (3.66-3.78)	4.79 (4.70-4.87)	<0.01	-0.95
NVUGIB	3.69 (3.62-3.75)	4.78 (4.69-4.87)	<0.01	-0.97
VUGIB	4.47 (4.17-4.77)	5.11 (4.51-5.71)	0.2	-0.48

## Discussion

This study demonstrated that patients admitted for nonvariceal UGIB to teaching hospitals had similar outcomes when compared to those admitted to non-teaching hospitals in terms of mortality, rate of in-hospital endoscopy, and early endoscopy within 24 hours. However, teaching hospitals had higher rates of in-hospital therapeutic endoscopy, length of stay, and subsequent charges compared to non-teaching hospitals. Patients admitted with variceal UGIB to teaching hospitals compared to non-teaching hospitals had similar outcome metrics in terms of mortality, rate of in-hospital endoscopy, early endoscopy within 24 hours, rate of endoscopic therapy, and total hospital charges. However, the length of stay was higher among those admitted to teaching hospitals.

A more recent systematic review and meta-analysis from 2014 showed no convincing evidence that outcomes differ when comparing teaching and non-teaching medicine services [3]. A closer examination of some studies for patients with all GI bleeding has found that teaching hospitals tend to have higher mortality, number of endoscopies, and likelihood of shock and mechanical ventilation [4]. Further inquiry into outcomes specifically for UGIB has once again shown contrasting results from the current literature when comparing teaching and non-teaching hospitals [13]. Asotibe et al. (2021) [5] performed a stratified analysis using the National Inpatient Sample database from 2016 and 2017. Results from that retrospective cohort study showed that patients with nonvariceal UGIB (n=94,900) had better in-hospital outcomes including mortality, median length of stay, and total hospital charges in non-teaching hospitals compared to teaching hospitals. Patel et al. (2020) [6] performed another retrospective cohort study regarding hospital teaching status and the outcomes of patients with variceal UGIB from 2008 to 2014 using the National Inpatient Sample database. Results from that retrospective cohort study showed that patients with variceal UGIB (n=58,362) had higher rates of mortality, length of stay, and costs in teaching hospitals compared to non-teaching hospitals. A possible explanation for this finding is that patients admitted to teaching hospitals compared to non-teaching hospitals were more likely to have decompensated liver cirrhosis with spontaneous bacterial peritonitis or hepatocellular carcinoma or refractory UGIB requiring rescue procedures such as portosystemic shunt.

The contrasting results from the current literature and prior studies regarding teaching hospital status and outcomes for UGIB warranted further inquiry. This study attempted to help with this discrepancy and included two groups of patients with UGIB: patients with either variceal or nonvariceal bleeding. For our study, the National Inpatient Sample database for the calendar year 2014 was used to compare outcomes in NVUGIB and VUGIB in teaching and non-teaching hospitals (combined NVUGIB and VUGIB: n=136,285). The strengths of this study are exemplified using multivariate analysis in both NVUGIB and VUGIB groups by looking at specific different outcomes metrics (mortality, length of stay, rate of endoscopy, early endoscopy < 24 hours, therapeutic endoscopy, PRBC transfusion, and charges) between the two groups in both teaching and non-teaching hospitals.

In addition, a univariate analysis was performed to identify potential confounders for age, race, sex, hospital region and size, insurance type, and admission in July to provide accurate adjusted odds ratios for mortality for both VUGIB and NVUGIB cohorts in teaching and non-teaching hospitals during this time period. Our study also used a novel approach that looks at both VUGIB and NVUGIB and compares outcome metrics for teaching and non-teaching hospitals during the same time period. The power of this study for NVUGIB was larger as it included 37,185 more patients, which serves as a plausible theory as to why our study outcomes differ from the findings of Asotibe et al. (2021) [5] for NVUGIB in terms of mortality.

While our study has a much lower power when comparing VUGIB (n=4200) to the findings of Patel et al. (2020) (n=58,362) [6], it should be noted that within the past 20 years, there have been significant advances in the management of portal hypertension. For instance, in 2013-2014, 80%-90% of transjugular intrahepatic portosystemic shunt (TIPS) procedures became elective with success rates greater than 97% [7]. A large-scale retrospective study in 2016 by Lee et al. (2016) [14] used the National Inpatient Sample database from 1998 to 2012 and looked for trends for inpatient death following TIPS procedures. Findings from this study showed that there was a significant decrease in mortality after 2005 with the introduction of covered stent grafts from 1998-2004 (13.15%) to 2005-2012 (11.50%). A second large-scale retrospective study by Mah et al. (2019) [15] compared mortality for patients who received TIPS at teaching versus non-teaching hospitals from 1998 to 2016 in Canada. Their results showed that 84.3% of TIPS procedures were performed in teaching hospitals compared to 15.7% in non-teaching hospitals. Their results also indicated that hospital teaching status was not associated with mortality despite adjusting for confounders and clustering. Current data supports the notion that mortality from VUGIB is in fact decreasing. While previous studies indicate that there is a difference in mortality between teaching and non-teaching hospitals for VUGIB, it should be noted that there are significant advances that have occurred in the management of variceal bleeding starting from 2005 onward, and the rates of TIPS procedures have significantly increased during this period with increasing success rates and primarily occurring at major academic centers. We suspect that this is one of the reasons why the rates of mortality in our study are similar for VUGIB in teaching and non-teaching hospitals [14]. Further prospective study is warranted to identify mortality rates for VUGIB when comparing teaching and non-teaching hospitals.

The secondary outcomes for NVUGIB included higher rates of in-hospital therapeutic endoscopy, length of stay, and subsequent charges at teaching hospitals compared to non-teaching hospitals. Our study had similar findings to those of Asotibe et al. (2021) [5], specifically when it comes to length of stay and charges. One plausible theory for this is that academic centers, which focus on teaching fellows, have more time constraints compared to solo providers at non-teaching hospitals who likely have greater turnover due to increased proficiency. In addition, the rate of therapeutic endoscopy was higher in teaching hospitals, which is a major factor when considering costs, equipment, and personnel. For this reason, patients admitted to teaching hospitals likely had greater length of stay and subsequent charges.

The secondary outcomes for VUGIB, including the rate of in-hospital endoscopy, early endoscopy within 24 hours, endoscopic therapy rate, and total hospital charges, were similar in teaching and non-teaching hospitals. Our study had some different findings from those of Patel et al. (2020) [6], specifically when it comes to costs. Interestingly, both studies found similar findings for increased length of stay at teaching compared to non-teaching hospitals. Our study also demonstrated that the rates of therapeutic endoscopy were similar in teaching and non-teaching hospitals. This is a major factor when considering costs and was likely one of the major reasons why our study found that costs were similar in this cohort at both teaching and non-teaching hospitals. There could be a multitude of factors for increased length of stay at teaching hospitals. We primarily hypothesize that many patients with variceal bleeding, especially those with acute instability, often get transferred to academic centers. These patients must deal with delays in transportation or transfer after undergoing intervention. Further study to characterize why length of stay is increased in this group is warranted.

There are some limitations to our study. As a retrospective cohort study, our results can only establish associations but not implicate causality. Our study also only included patients from the year 2014, which had less power especially when comparing the outcome metrics for VUGIB between teaching and non-teaching hospitals. The NIS database also reports data on hospitalization instead of individual patients; therefore, some patients who have been hospitalized multiple times can be accounted for multiple times. Lastly, our data does not have information describing the involvement of fellows or their training expertise.

## Conclusions

Current data regarding outcomes for patients with UGIB (variceal and nonvariceal) treated at teaching and non-teaching hospitals have demonstrated mixed results. Further investigation is warranted to see if there are differences in outcomes when comparing teaching to non-teaching hospitals for UGIB.
